# Obituary

**Published:** 1889-07

**Authors:** 


### Obituary.

Died June 7th at his home in Chillicothe, Ohio, of paralysis, Dr. F. H. Rehwinkel. Death occurred the second day after the attack.

A more extended notice will be given in the next number of the Register.

---

Died June 15th at his home in Wyoming, near Cincinnati, Dr. C. R. Taft, of cerebral paralysis, which was brought on by an affection contracted in the army during the late war. Further notice hereafter.

